# Deoxycholic acid inducing chronic atrophic gastritis with colonic mucosal lesion correlated to mucosal immune dysfunction in rats

**DOI:** 10.1038/s41598-024-66660-3

**Published:** 2024-07-09

**Authors:** Yuqin Cheng, Shuaishuai Wang, Wenfei Zhu, Zijing Xu, Ling Xiao, Jianping Wu, Yufen Meng, Junfeng Zhang, Chun Cheng

**Affiliations:** 1 School of Medicine, Nanjing University of Chinese Medicine, Nanjing, 210023 Jiangsu China; 2 Laboratory Animal Center, Nanjing University of Chinese Medicine, Nanjing, 210029 Jiangsu China

**Keywords:** Chronic atrophic gastritis, Colorectal mucosal lesion, Microbiota, Bile acids, Transcriptome, Microbiology, Diseases, Gastroenterology

## Abstract

The present study aimed to explore the underlying mechanism of bile reflux-inducing chronic atrophic gastritis (CAG) with colonic mucosal lesion. The rat model of CAG with colonic mucosal lesion was induced by free-drinking 20 mmol/L sodium deoxycholate to simulate bile reflux and 2% cold sodium salicylate for 12 weeks. In comparison to the control group, the model rats had increased abundances of *Bacteroidetes* and *Firmicutes* but had decreased abundances of *Proteobacteria* and *Fusobacterium*. Several gut bacteria with bile acids transformation ability were enriched in the model group, such as *Blautia*, *Phascolarctobacter*, and *Enterococcus*. The cytotoxic deoxycholic acid and lithocholic acid were significantly increased in the model group. Transcriptome analysis of colonic tissues presented that the down-regulated genes enriched in T cell receptor signaling pathway, antigen processing and presentation, Th17 cell differentiation, Th1 and Th2 cell differentiation, and intestinal immune network for IgA production in the model group. These results suggest that bile reflux-inducing CAG with colonic mucosal lesion accompanied by gut dysbacteriosis, mucosal immunocompromise, and increased gene expressions related to repair of intestinal mucosal injury.

## Introduction

Chronic atrophic gastritis (CAG) is a common gastrointestinal disease, whose pathological features present thinning of gastric mucosa, with or without intestinal metaplasia. The main manifestations of CAG include loss of appetite, post-prandial fullness, early satiety, epigastric pain, heartburn, and regurgitation. In addition, CAG is often accompanied by extra-gastrointestinal manifestations, such as pernicious anemia, paraesthesia, autoimmune thyroid disease, and so on^1^. Accumulating studies have documented that the disorders of digestive organs are accompanied by intestinal mucosal lesions, for example, severe acute pancreatitis (SAP) patients accompanied by intestinal mucosal barrier injury^2^. In the further SAP mice model by injecting intraperitoneally with caerulein (100 μg/kg), small intestinal villi were decreased and damaged with severe apoptosis cells and high levels of inflammatory cytokines including IL-1β, TNF-α, CXCL2, and MCP-1; the fecal *Firmicutes* / *Bacteroidetes* ratio was increased, especially the four bacterial genera (*Dubosiella*, *Desulfovibrio*, *Escherichia-Shigella*, and *Enterococcus*)^3^. In the liver fibrosis mice model induced by injecting intraperitoneally with CCl_4_ and olive oil (1:9, v/v)^4^, extensive edema and many infiltrated lymphocytes appeared in ileum mucosal layer, as well as villus height, villus width, and crypt depth were significantly decreased; the abundances of gut *Mucispirillum* and *Lactobacillus* were significantly increased, while the abundance of *Ruminococcus_1* was significantly decreased. Clinical study^5^ found that ursodeoxycholic acid (UDCA) could partially relieve gut dysbacteriosis in patients with primary biliary cholangitis, and reduced the abundances of gut *Haemophilus*, *Streptococcus*, and *Pseudomonas*. Similarly, CAG patients are also accompanied by intestinal manifestations, such as lack of strength during defecation, sticky defecation, and borborygmus^6^. These results suggested that non-intestinal diseases are often linked with intestinal mucosal lesions and gut dysbacteriosis, but the detailed molecular mechanism was still unclear.

Epidemiological investigations^7^ showed that CAG and/or intestinal metaplasia closely correlated with *Helicobacter pylori* infection, bile reflux, and host genetics. Among them, high-frequency bile reflux was related to an elevated risk of intestinal metaplasia^8^, indicating that bile reflux plays a key role in the occurrence and development of CAG. Bile acid (BA) is an important regulator in cholesterol metabolism, fat digestion, and other metabolic pathways. Primary BAs conjugate with glycine or taurine in the liver to form conjugated BAs, and the conjugated BAs accumulate in the gallbladder and release into the duodenum; about 95% of BAs are reabsorbed in the distal ileum, while the unabsorbed BAs serve as substrates for microbial metabolism and transformed to secondary BAs in the colon and rectum^9^. Clinical study has shown that bile reflux not only affects the gastric environment, but also alters the gastric microbiota, with increased diversity and richness of gastric microbiota, such as *Comamonas*, *Halomonas*, *Bradymonas*, *Pseudomonas*, *Marinobacter*, *Arthrobacter*, and *Shewanella*^10^. Furthermore, bile reflux also affected gut microbiota, decreased the abundances of *Bifidobacterium*, *Prevotella_2*, *Ruminococcus*, *Weissella*, *Neisseria*, and *Akkermansia*, and increased the abundances of *Alloprevotella*, *Prevotella_9*, *Parabacteroides*, and *Megamonas*^11^. These results suggested that bile reflux could distinctly change the gastrointestinal microbiota, and then damage the barrier function of intestinal mucosa.

Many studies^12^ have shown that BAs participate in intestinal mucosal immunity by influencing gut microbiota and leading to inflammatory bowel disease and colorectal cancer. Yoshimoto et al.^13^ found that obesity-inducing gut dysbacteriosis could promote the production of intestinal deoxycholic acid (DCA) and the development of liver cancer while blocking DCA or recovering gut microbiota could prevent the development of liver cancer. BAs have antimicrobial activity, conversely, the microbiota could chemically modify the BAs with four patterns: deconjugation, dehydroxylation, oxidation, and epimerization. Bile salt hydrolases (BSHs) can deconjugate both glycine and taurine from primary BAs and produce low toxic free BAs, whose genes are widely expressed in many bacteria, such as *Bacteroides*, *Bifidobacterium*, *Lactobacillus*, *Clostridium, Enterococcus*, and *Listeria*^14^. Joyce et al.^15^ demonstrated that gut dysbacteriosis and reduced deconjugation of primary BAs increased the risk of ulcerative colitis, Crohn’s disease, and irritable bowel syndrome. Moreover, Staley et al.^9^ found that *Clostridium scindens* with the 7α-dehydroxylase gene significantly increased fecal BAs, and the increased *Clostridium cluster XVIa* was observed in the gut of colorectal cancer patients. These results suggested that the crosstalk between BAs and gut microbiota plays an important role in the development of intestinal inflammatory diseases and colorectal cancer.

Based on the accidental discovery of colonic mucosal lesion in CAG rats induced by 20 mmol/L sodium deoxycholate freely drink combined with 2% cold sodium salicylate solution, the present study assumed that bile reflux led to gastrointestinal comorbidities. Thus, here combined the omics analysis with gut microbiota sequencing, fecal BAs targeted metabolomics, and colonic mucosal transcriptome, and aimed to explore the underlying mechanism of CAG with concomitant colonic mucosal lesion. The present data will provide new potential targets and strategies for clinical comprehensive prevention and treatment of colonic mucosal lesion in CAG.

## Materials and methods

### Animals

Specific-pathogen-free (SPF) male Sprague–Dawley (SD) rats (170 ± 20 g, 5–6 weeks old) were purchased from Zhejiang Weitong Lihua Experimental Animal Technology Co., Ltd. (Zhejiang, China). All the SD rats were fed with SPF-grade sterilized diet (XIETONG SHENGWU, SWS9102) and water, and were controlled ambient room temperature (20 ± 2 °C) and air humidity (50–70%). The study protocol was approved by the Animal Ethics and Protection Committee of Nanjing University of Chinese Medicine (No. 202102A004). All experimental procedures were conducted in conformity with the institutional guidelines issued by the IACUC of NUCM and the ARRIVE guidelines.

### Establishment of CAG rat with colonic mucosal lesion

The rats were randomly assigned to either the control or model group after one week of adaptive feeding. The model rats were treated with 20 mmol/L deoxycholate sodium (Beyotime Biotech. Inc., China, No. ST2049-500 g) and 2% cold sodium salicylate (Sigma, No. S3007) as previous description^16^. The control rats were fed with fodder, freely drank sterile water, and received 2 mL of sterile water every day. The general information was carefully recorded weekly, such as fecal particles, weight, and rat appearance (hair, mouth, and nose). After 12 weeks, the model was established successfully by histopathological examination.

### Sample collection and processing

All rats were fasted without water deprivation for 24 h, and anesthetized by isoflurane (RWD, R510-22) gas with an induction dose of 6 mL/h and a maintenance dose of 4.5 mL/h. After anesthetization, the rats were sacrificed by exsanguinating from the celiac artery. The stomach was separated, rinsed the stomach contents, and washed gently with normal saline water. Then, the gastric antrum tissue was fixed with 4% neutral paraformaldehyde. Three identical colon tissues were collected about 3 cm near the end of the cecum, and washed with sterile saline. One colon sample was fixed with 4% neutral paraformaldehyde, and the other two colon samples were quickly frozen in liquid nitrogen and stored at −80 °C. The feces were aseptically collected from the end of the colorectum, quickly frozen in liquid nitrogen, and stored at −80 °C.

### Histopathological examination

The gastric antrum tissues fixed with 4% paraformaldehyde were stained with hematoxylin–eosin (H–E). The colon tissues fixed with 4% paraformaldehyde were stained with H–E and Alcian Blue-periodic acid Schiff (AB-PAS). The pathological manifestations of the gastric antrum and colon tissue were observed under the microscope, such as tissue structure integrity and inflammatory infiltration.

### Analysis of fecal microbiota

Fecal microbiota was detected by 16S ribosomal RNA gene high-throughput gene sequencing. The total fecal DNA was extracted, and the specific amplification of the V3-V4 region of the bacteria 16S rDNA was amplified by polymerase chain reaction (PCR). The universal primers were 341F5′-CCTAYGGGRBGCASCAG-3 and 806R5′-GGACTACNNGGGTATCTAAT-3′. PCR products were detected and quantified using the QuantiFluor™-ST blue fluorescence quantification system (Promega), and were retrieved as the MiSeq PE library. The sequencing was completed using the Illumina PE250 platform by Shanghai Biozeron Biotechnology Co. Ltd (Shanghai, China). The valid data was obtained by QIIME (version 1.17). 97% similarity cluster analysis was performed to generate operational taxonomic units (OTUs) with Usearch software. The common bioinformatics analysis was conducted with Visual Genomics (Release 1, Shanghai Infinity Biotechnology Co., Ltd.) including microbial richness and diversity, microbial community structure, linear discriminant analysis (LDA), and PICRUSt function prediction analysis.

### Transcriptome analysis of colonic mucosa

The total RNA of colonic mucosa was extracted according to the standard protocol of the TRIzol Kit (Invitrogen, CA, USA). The purity and integrity of RNA were measured by Nanodrop 2000 Ultrafine Spectrophotometer (Thermo Fisher Scientific, USA) and Agilent 2100 Bioanalyzer (Agilent, USA), respectively. mRNA was enriched and reversed into cDNA with TruSeqTM RNA Sample Prep Kit (Illumina, USA), and cDNA was subjected to end repair. The cDNA library was amplified by bridge PCR, and high-throughput sequencing was performed using the Illumina NovaSeq 6000 platform.

In the RNA-seq analysis, gene expression levels were estimated by the number of clean reads mapped to genomic regions. According to the comparison of all samples to reference genomes, the value of FKPM (fragments per kilobase of the exon model per million mapped fragments) was calculated as the expression amount of a gene or transcript in the sample. Significant difference analysis was performed to screen differentially expressed genes (DEGs). The filter criteria of DEGs were FDR ≤ 0.05, and the absolute value of Log_2_ (fold change, FC) ≥ 1.00. The volcano plot presented the differential expression status of genes. Based on the DEGs, GO and KEGG pathway enrichment analyses were performed using the R package “ClusterProfiler”.

### Targeted metabolomics analysis of fecal bile acids

Vortex mixed fecal samples were centrifuged at 4000 rpm at 4 °C for 10 min. The supernatant was separated into an Eppendorf tube, and diluted to 10 and 1000 times, respectively. Then 100 μL of them were taken respectively and homogenized with 500 μL of acetonitrile/methanol (8:2), which contained mixed internal standards, and centrifuged at 12,000 rpm for 20 min to remove the protein. After that, the supernatant was dried up with a nitrogen blower. Then the precipitate was reconstituted with 100 μL of water/acetonitrile (8:2) with formic acid (0.1%) by well vortexing and centrifuging. Finally, the supernatant (2 μL) was injected into the ultra-high performance liquid chromatography coupled to tandem mass spectrometry (UHPLC-MS/MS) system for analysis.

The UHPLC-MS/MS system included AB Scienx ExionLCTM AD liquid chromatography column and AB Sciex QTRAP® 6500 + mass spectrometer. The sample was separated on the Waters ACQUITY UPLC BEH C18 column (2.1 × 100 mm, 1.7 μm) by setting the temperature at 50 °C. The mobile phase, consisting of 0.1% formic acid in water (solvent A) and acetonitrile (solvent B), was delivered at a flow rate of 0.30 mL/min. The solvent gradient was set as follows: 0 min, 80% A; 0.5 min, 80% A; 1.0 min,65% A; 2.5 min, 63% A; 4.1 min, 62% A; 5.0 min, 62% A; 6.0 min, 61% A; 6.5 min, 60% A; 8.5 min, 56% A;9.0 min, 55% A; 9.5 min, 48% A; 12.5 min, 35% A; 13.0 min, 0% A; 15.0 min, 0% A;15.1 min, 80% A,17.0 min 80% A. The BAs eluted from the chromatographic column were ionized by electrospray ionization (ESI) in negative-ion mode. The parameters were as follows: ion source temperature of 550 °C, ion source voltage of −4500 V, curtain gas of 30 psi, and atomization gas and auxiliary gas of 65 psi. Quantitative metabolite data were obtained by multiple response monitoring (MRM) for multivariate pattern recognition analysis and differential analysis.

### RT-qPCR verification

The total RNA of the colonic mucosa was extracted with TRI Reagent (Sigma, St. Louis, USA), and reversed transcribed into cDNA using PrimeScript RT kit (TaKaRa). Primers (Supplementary Table 1) were used for polymerase chain reaction (PCR), and the 20 μL PCR system included SYBR Green Mix 10 μL, primer 2 μL, sterile water 3 μL, and cDNA template 5 μL. Quantitative PCR (qPCR) amplification was performed for 35 cycles with β-actin as the internal reference. The expression level of each gene was measured three times, and calculated by the 2^-ΔΔCt^ method.

### Statistics

The data were analyzed using SPSS 26.0 (SPSS, inc.). The normally distributed metrological data were analyzed by Student's *t*-test and were presented as mean ± standard deviation (SDs). The non-normally distributed metrological data were analyzed by nonparametric tests. All tests were two-tailed, and *P* < 0.05 was regarded as statistically significant.

### Ethical approval

The animal study was reviewed and approved by the Animal Ethics Committee of Nanjing University of Chinese Medicine.

## Results

### Characteristics of CAG rats with colonic mucosal lesion

**Figure 1:**
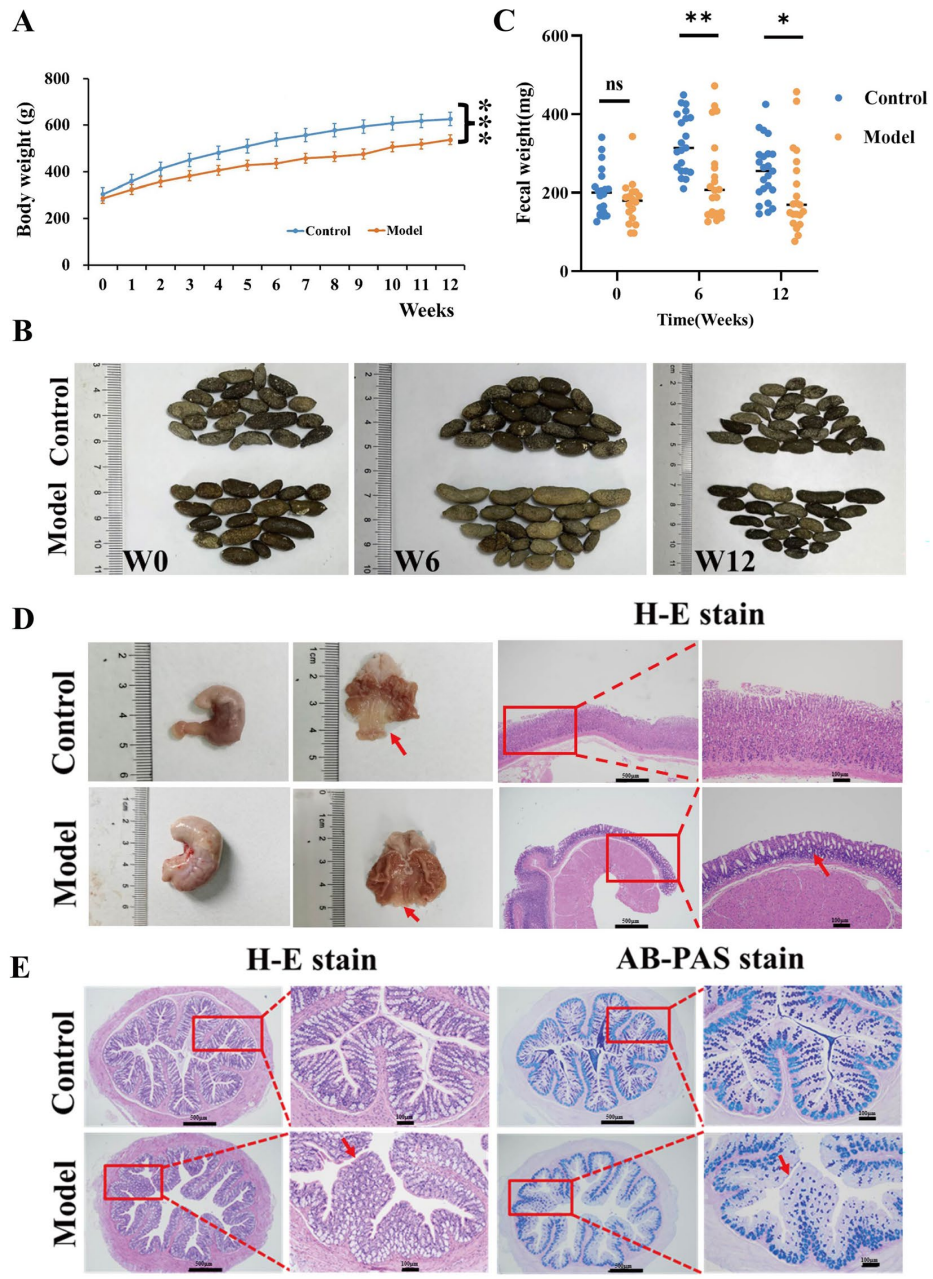
Occurrence of CAG with colonic mucosal lesion induced by sodium deoxycholate and sodium salicylate in rats. (**A**) The body weight of model group significantly became lower (*P* < 0.001) (Control, n = 6; Model, n = 6). (**B**) The fecal particles of model group became disproportionate. (**C**) The fecal weight of model group significantly became lower (*P* < 0.05). (**D**) Gastric morphology and H-E staining printed the occurrence of CAG. (**E**) H-E and AB-PAS staining showed colonic mucosal lesion.

The CAG rats with colonic mucosal lesion were successfully established using sodium deoxycholate and sodium salicylate for 12 weeks. Among them, the incidence of CAG was 75% (15/20), the incidence of colonic mucosal lesion in CAG rats was 40% (6/15), and the six CAG rats with colonic mucosal lesion were enrolled as model group in the present study (Fig. 1). The body weight of control rats increased evenly from 224.9 g to 626.8 g, however, the body weight of model rats increased slowly and was significantly lower than that of control rats (*P* < 0.001) (Fig. 1A). During the molding process, the fecal size of model group became different from control group; at 12th week, the fecal particle of model group became disproportionate (Fig. 1B) and significantly light-weighted (*P* < 0.05) (Fig. 1C). Compared to the control group, the stomachs of model group were obviously distended, the gastric mucosae were pale, the folds became reduced and thickened, and the glands were obviously reduced and irregularly arranged. Meanwhile, the gastric fovea lengthened, twisted, and even dendritically extended into the lamina propria. These results indicated that the CAG rat model was successfully established (Fig. 1D).

H-E staining showed that the colonic mucosal structure of control group was integrated, and the epithelial cells were arranged in an orderly manner, without bleeding points, erosions, or ulcers in the submucosae. However, the colonic submucosae of model group were disordered with bleeding spots, shortened or disappeared intestinal villi, and a large number of lymphocyte infiltration. The characteristic acid mucin secreted from intestinal goblet cells was stained blue by AB-PAS staining, and the reduction of blue stain was observed in the model group (Fig. 1E). The results indicated that the colonic mucosal lesion occurred in CAG rats.

### Alteration of gut microbiota in CAG rats with colonic mucosal lesion

**Figure 2:**
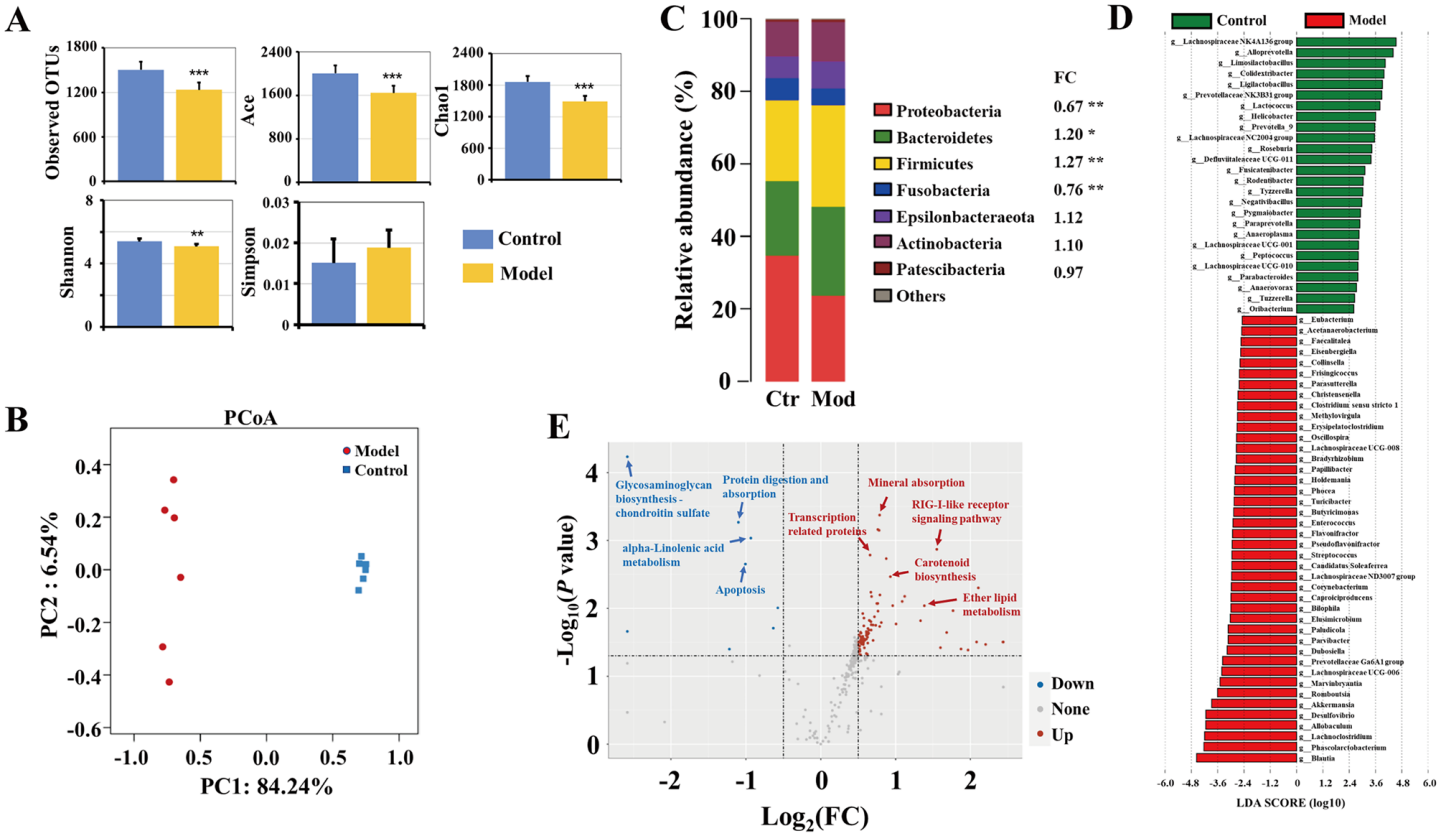
Alteration of gut microbiota in CAG rats with colonic mucosal lesion. (**A**) Alpha diversity analysis of gut microbiota (n = 6, data expressed as mean ± SD, and Mann–Whitney *U* test was used for different analysis, *P < 0.05, **P < 0.01, ***P < 0.001). (**B**) PCoA analysis presented the distinct alteration of gut microbiota between control and model groups. (**C**) The dominant community of gut microbiota was displayed at the phylum level. (**D**) LDA analysis presented the marked fecal genera of CAG with colonic mucosal lesion. (**E**) Volcano plot presented the different gut microbiota predictive functions, red points indicated up-regulated predictive signaling pathways, while blue points indicated down-regulated predictive signaling pathways.

Based on 16S rDNA high-throughput sequencing, the gut microbiota between model and control rats was significantly different (Fig. 2). Compared with the control group, the richness index (observed OTUs, Ace, Chao1) and diversity index (Shannon) of the gut microbiota in model group were significantly reduced (*P* < 0.01) (Fig. 2A). The principal co-ordinates analysis (PCoA) based on OTUs showed that the composition of gut microbiota in model group was distinctly different from control group (Fig. 2B). At the phylum level, the abundances of *Bacteroidetes* and *Firmicutes* were significantly increased (*P* < 0.05), while *Proteobacteria* and *Fusobacteria* were significantly decreased (*P* < 0.01) in model group (Fig. 2C).

LDA screened 146 significantly different bacterial taxa. Among them, 42 bacterial genera were enriched in model group, such as *Blautia* (9.73%), *Phascolarctobacterium* (3.67%), *Lachnoclostridium* (3.49%), *Desulfovibrio* (3.18%), *Allobaculum* (2.86%), *Akkermansia* (2.16%), *Romboutsia* (1.42%), and *Enterococcus* (0.0228%). Meanwhile, 26 bacterial genera were enriched in control group, such as *Prevotella_9* (1.59%), *Colidextribacter* (1.36%), *Limosilactobacillus* (0.95%), *Lachnospiraceae NK4A136 group* (0.83%), *Alloprevotella* (0.62%), *Lactococcus* (0.51%), *Helicobacter* (0.34%), *Prevotelaceae NK3B31 group* (0.20%), and *Ligilactobacillus* (0.19%) (Fig. 2D).

PICRUST was conducted to predict the KEGG function of gut microbiota. Here FDR ≤ 0.05 and |Log_2_ (FC)|≥ 0.5 as standards to screen the differential predictive function. In comparison to control group, 90 predictive functions significantly increased and eight predictive functions significantly decreased in model group. According to the *P* value, the most noteworthy predictive functions included five up-regulated signaling pathways (mineral absorption, RIG-I-like receptor signaling pathway, transcription-related proteins, carotenoid biosynthesis, and ether lipid metabolism) and four down-regulated signaling pathways (glycosaminoglycan biosynthesis-chondroitin sulfate, protein digestion and absorption, alpha-linolenic acid metabolism, and apoptosis) (Fig. 2E). The results suggested that bile reflex-inducing CAG went along with gut dysbacteriosis.

### Transcriptome analysis of colonic mucosa in CAG rats with colonic mucosal lesion

**Figure 3:**
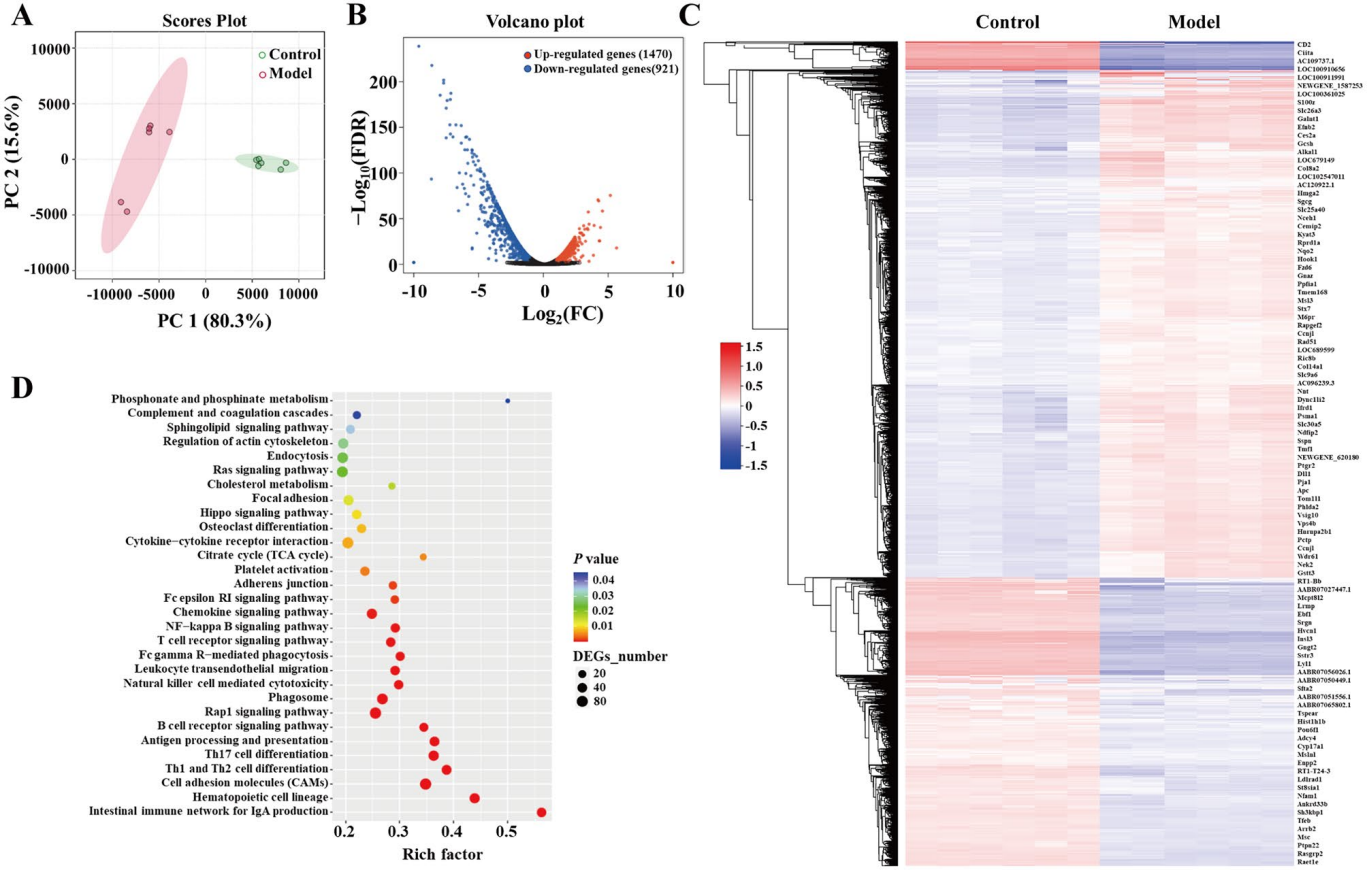
Alteration of gene expressive patterns in the colon of CAG with colonic mucosal lesion. (**A**) PCA presented the different gene expressive patterns between the model and control groups. (**B**) The volcano plot exhibited the DEGs of model group based on control group. (**C**) Hierarchical clustering heatmap displayed the top 100 DEGs (by R-Project 4.1.1). (**D**) Enrichment analysis of KEGG^17–19^ functions was conducted by the DEGs.

The predictive function of gut microbiota showed that colonic mucosal lesion might correlated to the transcriptional alteration of colonic mucosa. This study profiled the gene expression of colonic mucosal lesion tissues using RNA-sequencing technology. The principal component analysis (PCA) revealed the distinct gene expressive patterns between the model and control groups (Fig. 3A). According to the criteria of FDR ≤ 0.05 and |Log_2_ (FC)|≥ 1, there were 1470 up-regulated genes and 921 down-regulated genes in the model group (Fig. 3B). The hierarchical clustering algorithm displayed 100 genes with the most significant differences. Among them, the notable two up-regulated genes were Hnrnpa2b1 (heterogenous nuclear ribonucleoprotein A2/B1) and APC (adenomatous polyposis coli), which associated with cell proliferation; whereas Ciita (the class II major histocompatibility complex transistor), RT1-Bb (RT1 class II, RT1 class II, locus Bb), and Lrmp (lymphoid-restricted membrane protein), which associated with immune function, were significantly down-regulated (Fig. 3C). In addition, enrichment analysis of KEGG functions showed that the down-regulated DEGs were mainly enriched in the signaling pathways related immune response, such as complement and coagulation cascades, Fc epsilon RI signaling pathway, T cell receptor signaling pathway, Fc gamma R-mediated phagocytosis, leukocyte transendothelial migration, natural killer cell-mediated cytotoxicity, B cell receptor signaling pathway, antigen processing and presentation, Th17 cell differentiation, Th1 and Th2 cell differentiation, and the intestinal immune network for IgA production, as well as cholesterol metabolism pathways (Fig. 3D). The data suggested that CAG with colonic mucosal lesion was closely related to mucosal immune dysfunction.

### Targeted metabolome of fecal BAs in the CAG rats with colonic mucosal lesion

**Figure 4:**
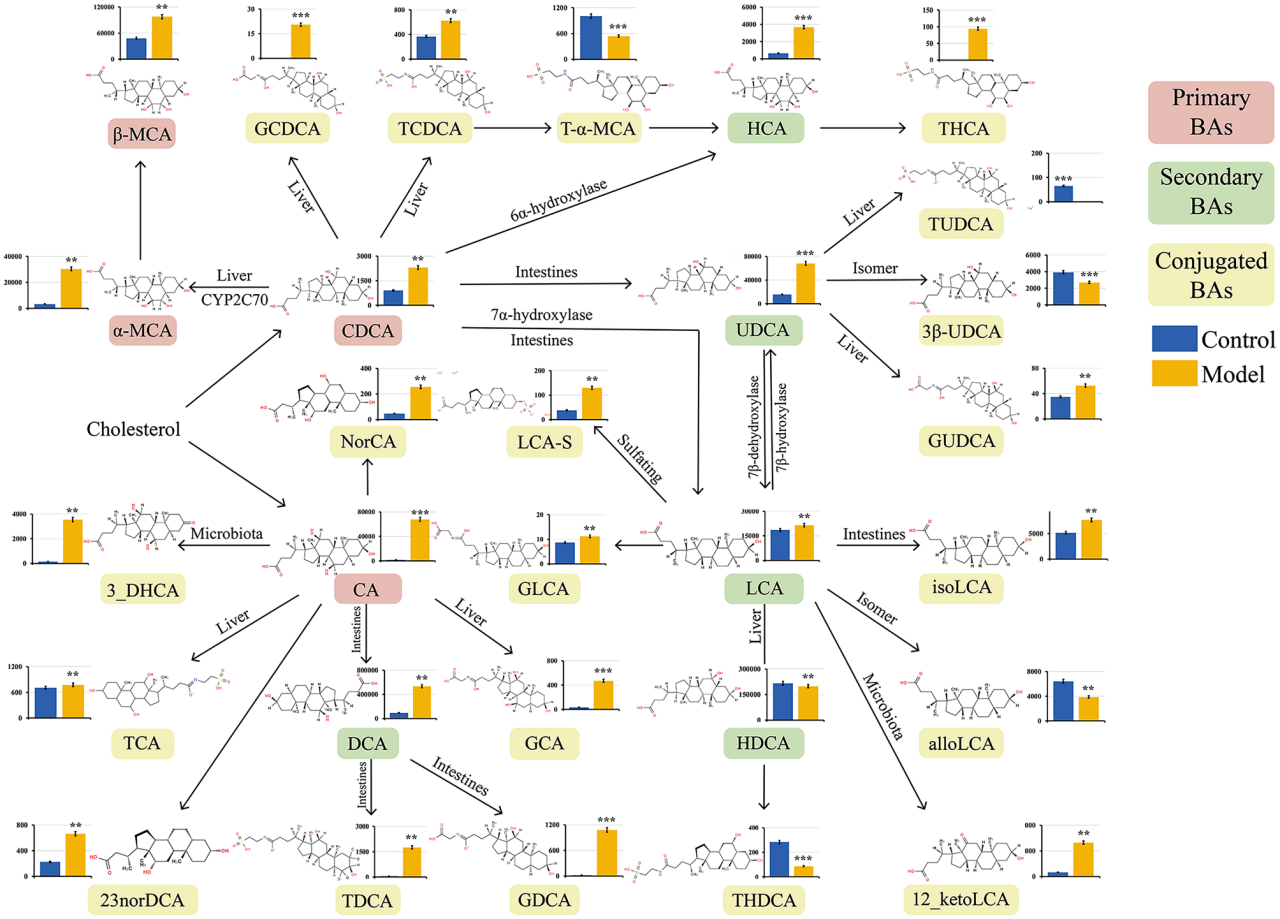
Quantitative analysis of fecal BAs in the CAG rats with colonic mucosal lesion. Nonparametric test analysis was conducted to analyze the differences between control and model groups, *P < 0.05, **P < 0.01, ***P < 0.001. The unit of BA’s concentration is ng/mL. β-MCA, β-muricholic acid; GCDCA, glycochenodeoxycholic acid; TCDCA, taurochenodeoxycholic acid; T-α-MCA, tauro-α-muricholic acid; HCA, hyocholic acid; THCA, taurohyocholic acid; TUDCA, tauroursodeoxycholic acid; α-MCA, α-Muricholic acid; CDCA, chenodeoxycholic acid; UDCA, ursodeoxycholic acid; 3β-UDCA, 3β-ursodeoxycholic acid; NorCA, 23-norcholic acid; LCA-S, lithocholic acid sulfate; GUDCA, glycoursodeoxycholic acid; 3-DHCA, 3-dehydrocholic acid; CA, cholic acid; GLCA, glycolithocholic acid; LCA, lithocholic acid; isoLCA, isolithocholic acid; TCA, taurocholic acid; DCA, deoxycholic acid; GCA, glycocholic acid; HDCA, hyodeoxycholic acid; alloLCA, allolithocholic acid; 23norDCA, 23-nordeoxycholic acid; TDCA, taurodeoxycholic acid; GDCA, glycodeoxycholic acid; THDCA, taurohyodeoxycholic acid; 12-ketoLCA, 12-ketolithocholic acid.

Numerous studies have revealed that BAs could shape the gut microbiota, the gut microbiota participates in BAs metabolism, and vice versa. The gut microbiota could modify the BAs participate in extensive physiological regulation, and even trigger the immune responses and induce chronic inflammation^20^. Generally, BAs are classified into primary BAs, secondary BAs, and conjugated BAs^21^, here analyzed 33 fecal BAs to explore certain BAs affecting the occurrence of CAG with colonic mucosal lesion. The results showed that 29 fecal BAs were detected in the fecal samples, and all of them were significantly different between the control and model groups (*P* < 0.05), whose transformational relationship was presented in Fig. 4. Firstly, five primary BAs were significantly up-regulated in model group, including chenodeoxycholic acid (CDCA), cholic acid (CA), α-muricholic acid (α-MCA), β-muricholic acid (β-MCA), and ursodeoxycholic acid (UDCA). About the four secondary BAs, three up-regulated BAs included DCA, lithocholic acid (LCA), and hydroxycholic acid (HCA); while hyodeoxycholic acid (HDCA) was significantly down-regulated. About the 19 conjugated BAs, comparison to control group, 14 fecal BAs were significantly increased in model group, such as glycolchenodeoxycholic acid (GCDCA), taurocholic acid (THCA), taurocholic acid (TDCA), glycol deoxycholic acid (GDCA), and 12-ketolithocholic acid (12-ketoLCA); however, five conjugated BAs were significantly decreased in model group, including tauro-alpha-muricholic acid (T-α-MCA), 3β-ursodeoxycholic acid (3β-UDCA), taurosodeoxycholic acid (TUDCA), taurosodeoxycholic acid sodium (THDCA), and allocholic acid (alloLCA).

Interestingly, the primary BAs (CDCA, CA) and secondary BAs (DCA, LCA), which occupy a core position in the BAs transformation network, were significantly increased in model group. Since here used DCA as a model inducer, the fecal TDCA and GDCA were significantly increased in model group, indicating that gut microbiota was involved in the modification and transformation of DCA.

### Association analysis among gut microbiota, fecal BAs, and gene expression

**Figure 5:**
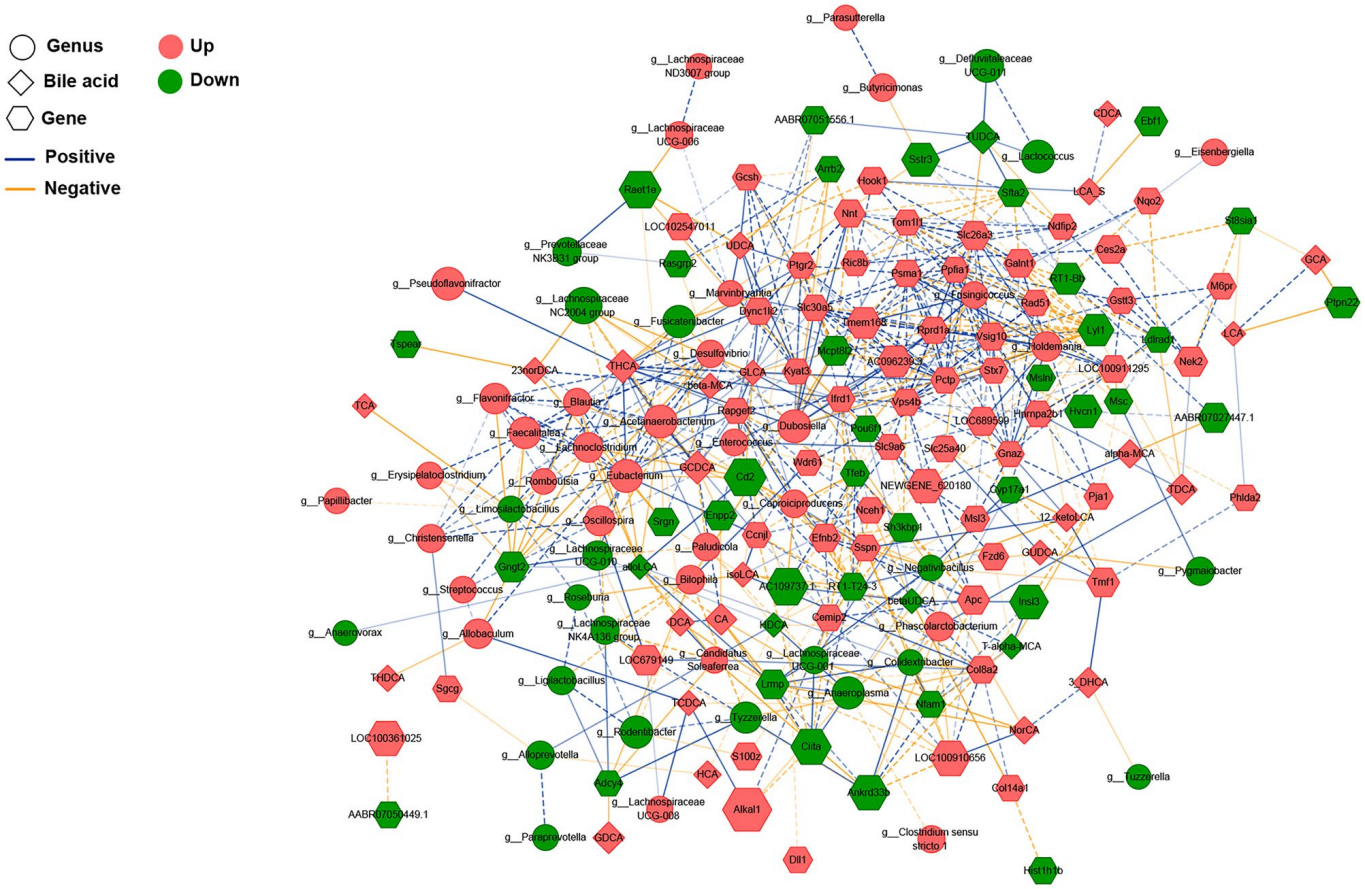
Correlated network of gut microbiota, fecal BAs, and colonic mucosal expression genes. Based on the 53 differential genera, 29 fecal BAs, and 100 colonic DEGs, Spearman’s correlation analysis was conducted to build a correlation network with the absolute value of correlation coefficient (*r*) greater than 0.9 and *P* value less than 0.05. The round nodes represent the bacterial genera, the diamond nodes represent fecal BAs, and the hexagonal nodes represent the colonic DEGs. The size of the node represents the quantitative ratio of model group to control group. In comparison to control group, the red nodes mean up-regulated, and the green nodes mean down-regulated. The blue and yellow lines refer to positive and negative correlations, respectively. The dotted line represents the internal correlation in each attribute, while the solid line represents the correlation between different attributes. As regards the color of the line, the darker indicates a greater correlation coefficient and the lighter indicates a smaller correlation coefficient.

To explore the underlying mechanism of the development of colonic mucosal lesion in the CAG rats, Spearman’s correlation analysis was performed to present the association among gut microbiota, fecal BAs, and colonic mucosal expression genes. The correlation network included 53 differential genera, 29 fecal BAs, and 100 mucosal DEGs (Fig. 5). CA could be transformed into DCA by certain bacteria and had significantly negative correlations with genus *Anaeroplasma*, Ciita, and Enpp2 (Ectonucleotide Pyrophosphatase/Phosphodiesterase 2) (*r* = −0.912, −0.923, −0.909), which indicated that bile stagnation might happen in the CAG rat with colonic mucosal lesion. Since Ciita plays a key role in the MHC-II-mediated antigen presentation pathway, the high level of fecal CA might inhibit the function of antigen presentation in the colonic mucosa.

TDCA, a conjugated BA originated from gut DCA, was significantly positively correlated with *Phascolarctobacterium*, Hnrnpa2b1, and Nek2 (NIMA related kinase 2) (*r* = 0.916, 0.909, 0.902), but was negatively correlated with RT1-Bb (RT1 class II, locus Bb) and Ldlrad1 (low density lipoprotein receptor class A domain containing 1) (*r* = −0.902, −0.909). *Phascolarctobacterium* was a dominant bacterium in the gut of model rats, indicating its involvement in the transformation and metabolism of TDCA. In addition, DCA could be transformed into GDCA by gut microbiota, and GDCA had a significantly negative correlation with Adcy4 (*r* = −0.916), suggesting that DCA might inhibit the generation of cyclic adenosine monophosphate in mucosal cells through GDCA, and reduce the capacity of damage resistance in the colonic mucosa. The above results indicated that the colonic mucosal lesion was linked closely with the alteration of gut dysbacteriosis-inducing BAs spectrum.

### Verification of key genes of signaling pathways related to CAG with colonic mucosal lesion

Here focused on the 90 DEGs enriched in 12 key signaling pathways to screen the key molecules in the development of bile reflux-inducing CAG rats with colonic mucosal lesion. Firstly, correlation analysis between the DEGs and fecal BAs (Supplementary Fig. 1) showed that DCA was significantly positively correlated with Cd3d (T-cell surface glycoprotein CD3 delta chain precursor) and Ciita (*r* = −0.839, −0.86, *P* < 0.01). Cd3d is a key molecule in the TCR signaling pathway, and Ciita is a key molecule in the antigen processing and presentation signaling pathway. In the colon of model rats, the expressions of Cd3e and Cd3g in the TCR signaling pathway were significantly decreased, and the expressions of 21 genes involved in antigen processing and presentation signaling pathway were significantly decreased, especially Ciita. In addition, DCA was significantly negatively correlated with most genes in the intestinal immune network for IgA production pathway (*P* < 0.01). RT1-Bb was broadly involved in antigen processing and presentation, Th17 cell differentiation, Th1/Th2 cell differentiation, and intestinal immune network for the IgA production pathway. These results indicated that high levels of fecal DCA and TDCA could inhibit the mucosal immune function, including antigen processing and presentation, Th17 cell differentiation, SIgA synthesis, and secretion.

Secondly, correlation analysis between the DEGs and bacterial genera (Supplementary Fig. 2) showed that *Candidatus Soleaferrea*, a key bacterium responding to DCA, had a significantly negative correlation with Cd3d (*r* = −0.874, *P* < 0.01) and a significantly positive correlation with Map2k1 (mitogen-activated protein kinase 1) (*r* = 0.832, *P* < 0.01). Cd3d is a key molecule in the TCR signaling pathway, Th17 cell differentiation, and Th1/Th2 cell differentiation signaling pathway, and Map2k1 is a key molecule in the FcεRI signaling pathway, TCR signaling pathway, Fcγ receptor-mediated phagocytosis, natural killer cell-mediated cytotoxicity, and B cell receptor signaling pathway. These results suggested that DCA might inhibit colonic T cell immune response function by inducing intestinal microbiota imbalance. Moreover, *Phascolarctobacterium*, a key bacterium responding to TDCA, was significantly negatively correlated with Cxcr4 (C-X-C chemokine receptor type 4) (*r* = −0.825, *P* < 0.01). Cxcr4 is a key molecule in the intestinal immune network for IgA production and white blood cell transdermal migration signaling pathway. These results suggested that DCA promoted the proliferation of *Phascolarctobacterium* through TDCA, inhibited intestinal IgA production and white blood cell endothelial migration, and promoted colonic mucosal inflammation. Similarly, in the colon of model rats, the expressions of Lck (proto-oncogene tyrosine-protein kinase LCK), Cxcr4, and Ciita were significantly down-regulated, which were positively correlated with *Anaeroplasma*. These results further confirmed that CAG with colonic mucosal lesion might be related to DCA-inducing gut dysbacteriosis. In addition, DCA was significantly positively correlated with Lrmp (a tumor promotion gene), and TDCA was significantly positively correlated with Hnrnpa2b1. The previous study found that Hnrnpa2b1 was related to the changes in immune cell infiltration, spliceosomes, and cell cycle signals^22^. These results suggested that the excessive BAs (DCA and its derivatives TDCA) altered gut microbiota and induced inflammatory colonic mucosal lesion, which further promoted continuous inflammatory lesion and even cancerization by weakening the colonic mucosal immune signaling pathways.

**Figure 6:**
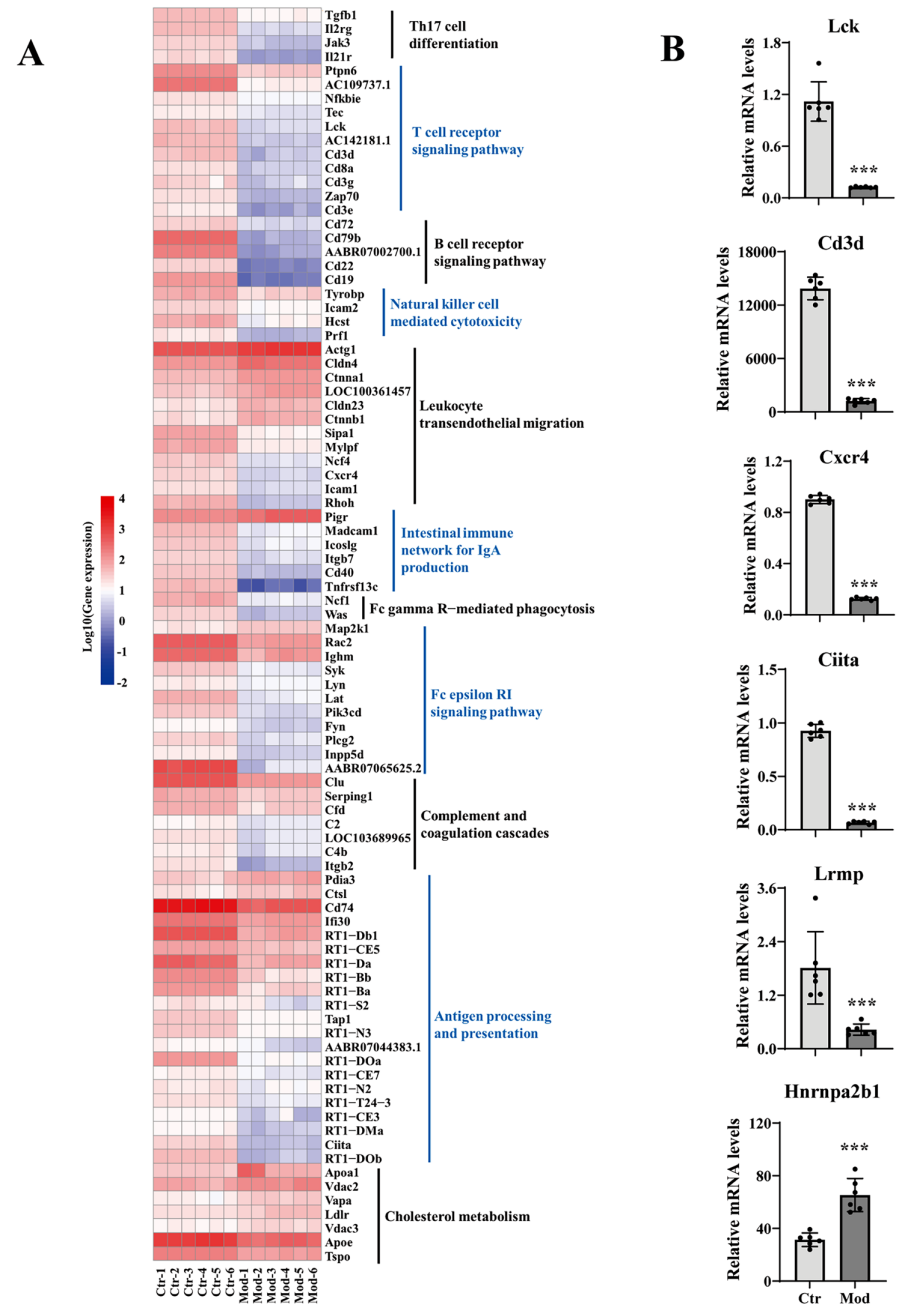
Verification of six key genes in the signaling pathway related to CAG with colonic mucosal lesion. (**A**) Heatmap presented the expressive levels of the genes in the eleven signaling pathways between control and model groups (by R-Project 4.1.1). (**B**) Verification of six genes in gene expression profiles by RT-qPCR, ***P < 0.001.

Finally, the four down-regulated signaling pathways were considered critical mechanisms, including antigen processing and presentation, TCR signaling pathway, IgA production intestinal immune network for IgA production, and leukocyte transendothelial migration (Fig. 6A). And RT-qPCR verified the expression levels of six key genes, including Lrmp, Hnrnpa2b1, Cd3d, Lck, Cxcr4, and Ciita. Among them, the expressions of Ciita, Cxcr4, Lrmp, Lck, and Cd3d were significantly decreased (*P* < 0.001), while the expression of Hnrnpa2b1 was significantly increased (*P* < 0.001), which was consistent with the results of the colonic transcriptome (Fig. 6B).

## Discussion

BAs are the most important emulsifier for fat digestion and absorption, and are considered as paracrine and endocrine signal molecules. They activate many nuclear receptors including farnesoid X receptor (FXR), pregnane X receptor (PXR), chimeric antigen receptor (CAR), vitamin D receptor (VDR), and Takeda G protein-coupled receptor 5 (TGR5). The activated receptors stimulate various kinase pathways promote the phosphorylation of histone regulatory proteins, and regulate the gene expression involved in integrated metabolism. When the BAs homeostasis was broken, they led to a series of metabolic diseases, such as cholestatic liver diseases, inflammatory bowel disease, obesity, and diabetes^23–25^. Xu et al.^26^ found that a diet with 0.2% DCA could decrease the expression of FXR protein in the ileum of mice, and the intestinal mucosa presented edema and thickening, crypt deformation, with a large number of lymphocyte and neutrophil infiltration. The present study observed colonic mucosal lesions in CAG rats induced by free-drinking DCA and found an abnormally fecal BA spectrum. Previous report found that fecal BAs had many functions, including activating intracellular adenylate cyclase, increasing intestinal mucosal permeability, promoting intestinal electrolyte and water secretion, and stimulating colonic motility and defecation^27^. Thus, Fan et al.^28^ believed that BAs were "physiological laxatives" because the slow transit constipation patients are often accompanied by abnormal BAs metabolism and dysbacteriosis. Alemi et al.^29^ found that the liposoluble BAs (DCA and LCA) could over-activate TGR5 in the intestinal epithelium to promote colon motility, and Alemi et al.^29^ found that DCA promoted colonel peristalsis by promoting the releasement of 5-HT and expression of calcitonin gene-related peptide (CGRP) via TGR5 signaling pathway in the intestinal chromaffin cells. Sagar et al.^30^ conducted a clinical study in the diarrhea patients with irritable bowel syndrome, and found that the fecal DCA and LCA had much higher concentrations with 4670 nmol/g and 1240 nmol/g, respectively. These evidences might partially interpret the disproportionate size and lighter fecal particles in the present model rats with increased secondary BAs (DCA and LCA). These results suggested that abnormal BAs metabolism altered the fecal formation.

On the other hand, certain BAs had beneficial functions. Pi et al.^31^ found that HDCA could alleviate the pathological manifestations of DSS-induced colitis mice, such as weight loss, colon shortening, and mucosal epithelial destruction; pro-inflammatory cytokines (IL-1β, IL-6, and TNF-α) in the colon were down-regulated, suggesting that HDCA had a protective effect against colitis. Pike et al.^32^ found that TUDCA was the most potent inhibitor of caspase-3/7 activation, and inhibited Caco-2 cell apoptosis induced by *Clostridium difficile* toxins. He et al.^33^ found that THDCA could decrease the serum levels of TNF-α and IL-6, the activity of myeloperoxidase (MPO), and the expression of COX-2 protein in colitis mice induced by intrarectal injection of trinitrobenzene sulfonic acid (TNBS), suggesting that THDCA had a protective effect against ulcerative colitis. In our study, the CAG with colonic mucosal lesion went along with decreased fecal HDCA, THDCA, and TUDCA. These results suggested that the abatement of beneficial BAs played a key role in the development of colonic mucosal lesion.

Mountain evidence showed that gut microbiota got extensively involved in the biotransformation and enterohepatic cycle of BAs^34^. In this study, the abundances of *Enterococcus* and *Blautia* related to BAs metabolism were significantly increased. A previous study^35^ found that the gene of BSH, an important metabolic enzyme, is distributed popularly in the gut *Enterococcus* and *Bacteroides*. Since BSHs could catalyze the deconjugation of primary BAs to free venenous BAs, here observed the high abundance of *Enterococcus* in the gut of model rats, which might convert the conjugated BAs into free BAs, such as CA and CDCA. *Blautia* occupies the gene of 7α-dehydroxygenase, which catalyzes the conversation of primary BAs into secondary BAs (DCA and LCA)^36^. Clinical studies^37^ found that a high level of fecal DCA was observed in patients with ulcerative colitis, atypical hyperplasia, or colorectal cancer, suggesting that high DCA may be a risk factor for colorectal cancer. Nishino et al.^38^ found an increased abundance of *Blautia* in the cecal and colon endoscopic brush samples from patients with ulcerative colitis. Similarly, here also found an enriched *Blautia* in the gut of model rats. The above data indicated that the amount of toxic DCA and LCA derived from bacterial transformation led to colonic mucosal lesion in the CAG rats.

The BAs not only shape the gut microbiota, but also directly function with the intestinal mucosal epithelial / immune cells, participating in the maintenance of intestinal mucosal homeostasis, damage and repair. Abnormal BAs metabolism play a key role in the development of inflammatory bowel disease^39^. Zeng et al.^40^ investigated the damaging effect of DCA on the cell adhesion structures of Caco-2 cells and found that DCA could inhibit ERK signaling pathways, meanwhile, the mRNA levels of 23 mucosal barrier-related genes were significantly decreased, including integrin subunit beta 2 (ITGB2), caveolin 1, cadherin 1, and claudin 1. The significantly decreased expression of ITGB2 in model rats indicated that excessive DCA caused abnormal BAs metabolism and disrupted the colonic mucosal barrier.

A recent study^41^ showed that some gut bacteria could produce 3α / 3β-hydroxysteroid dehydrogenase (3α/3β-HSDH) to convert LCA into isoLCA, and isoLCA could inhibit the differentiation and function of Th17 cells by directly inhibiting the expression of RORγt (retinoic acid receptor-related orphan nuclear receptor γt) in CD4^+^T cells, triggering the occurrence of inflammatory intestinal mucosal lesions. Liang et al.^42^ found that Th17 cells could co-express IL-17α and IL-22 under the stimulation of pro-inflammatory cytokines (TGF-β and IL-6), and IL-22 could induce epithelial cells to secrete antimicrobial peptides and mucins. In the feces of model rats, our data observed a higher level of isoLCA, but lower expressive levels of IL-22, antimicrobial peptide Reg3g (recombinant regenerating islet derived protein 3 gamma), and Th17 differentiation-related genes (Tgfb1, IL2rg, Jak3, IL21r) in colonic mucosa. Thus, here boldly speculated that redundant isoLCA inhibited the Th17 cell differentiation and function, and caused colonic mucosal lesion in the bile reflex-inducing CAG.

The down-regulated genes in the colonic mucosa of model rats were mainly enriched in immune-related pathways, such as Lrmp, Cxcr4, Ciita, Cd3d, and Lck. For example, Lrmp, encoding 539 amino acid proteins, could effectively transport COOH-terminal antigenic peptides to MHC I molecules and enhance the antigen presentation function in many kinds of cells, and its down-regulated expression was observed in several malignant tumors including colon cancer^43^ and lung cancer^44^. Throughout the MHC II-mediated antigen presentation pathway, Cxcr4 could promote the formation and stability of immunological synapses, and enhance the interaction between antigen-presenting cells (APCs) and T cells^45^. Ciita is a member of MHC II molecules on the surface of professional APCs and assists in presenting exogenous antigenic peptides to the TCR of CD4^+^ T cells^46^. CD3-TCR complex receives the extracellular antigen signals and transfers into intracellular cytoplasm via CD3 molecules (CD3D, CD3E, CD3G, and CD3Z) and Src family tyrosine kinase Lck protein^47^. These down-regulated molecules indicated that antigen presentation and T cell immune response were impaired in the colonic mucosa of the CAG rats with colonic mucosal lesion.

Previous studies found that DCA could not only disrupt the mucosal barriers but also promote intestinal tumorigenesis^48,49^. Hnrnpa2b1, an N6-methyladenosine (m6a) reader, can recognize pathogen DNA and mediate innate immune response, expressing highly in pan cancerous such as lung cancer, gastric cancer, and liver cancer, which is associated with poor prognosis^22^. Liu et al.^50^ found that MIR100HG interacted with Hnrnpa2b1 to maintain TCF7L2 mRNA stability, then, TCF7L2 enhanced the activity of the Wnt/β-catenin signaling pathway, and promoted epithelial-to-mesenchymal transition and the invasion of colorectal cancer. Tang et al.^51^ found that highly expressed Hnrnpa2b1 promoted the proliferation of colon cancer cells by activating the ERK/MAPK signaling. These evidence suggest that excessive DCA promote the gene expressions related to proliferation of intestinal epithelial cells.

**Figure 7:**
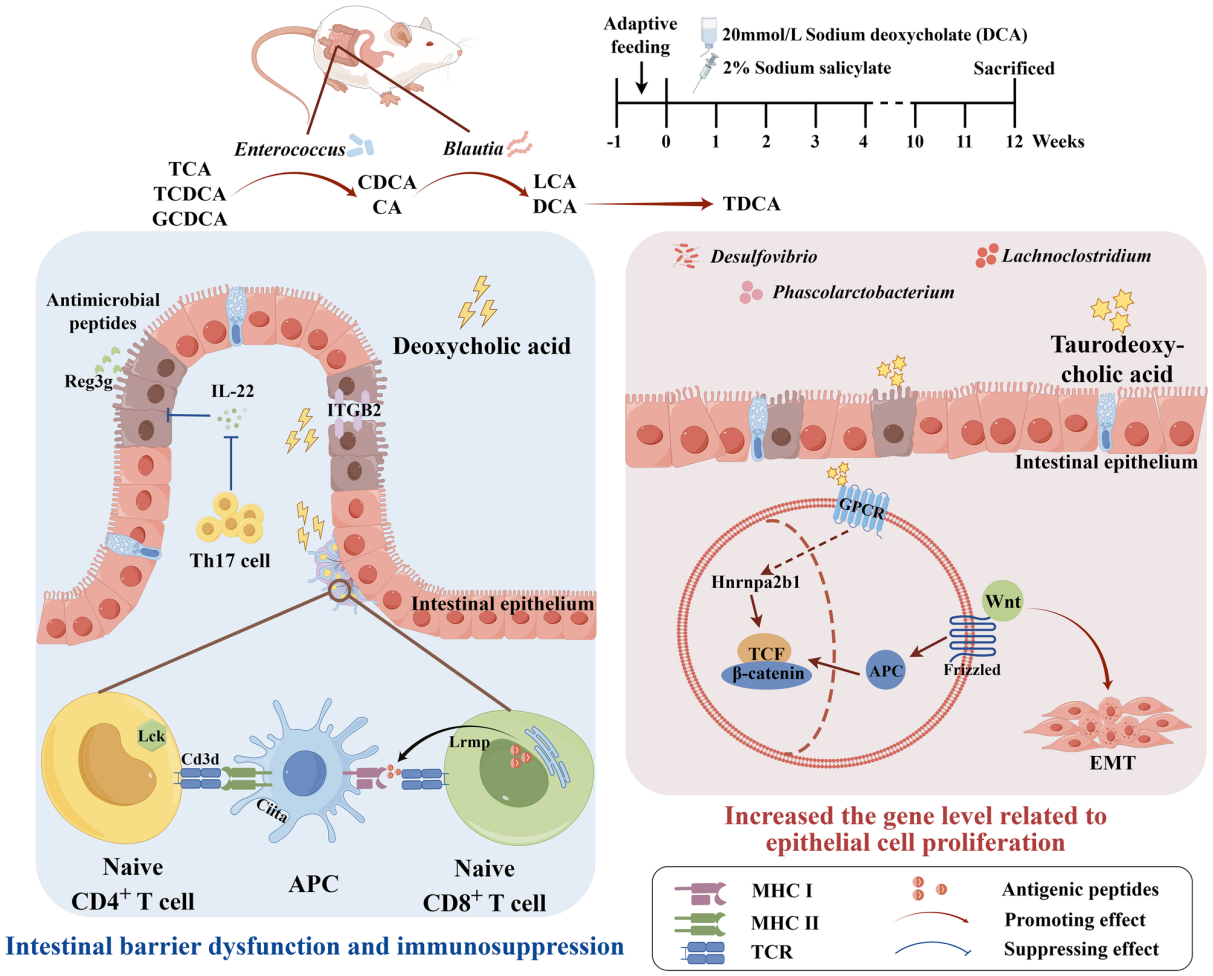
Research Overview. When the CAG rats with colonic mucosal lesion were successfully induced by the combination of 20 mmol/L sodium deoxycholate (DCA) and 2% sodium salicylate for 12 weeks, the persistent and complex interactions occurred among the abnormal BAs metabolism, gut microbiota, and colonic mucosal gene expression in the intestinal tract. Firstly, excessive sodium deoxycholate entered the intestinal tract and promoted *Enterococcus* and *Blautia*, which involved BAs transformation and caused significant elevation of most gut BAs, especially fecal DCA and LCA. Secondly, high levels of fecal DCA inhibited the expression of ITGB2 mRNA in colonic mucosa and disrupted the connections of intestinal epithelial cells. Furthermore, the expressions of Ciita, Cd3d, Lck, and Lrmp, related to antigen presentation and T cell activation, were significantly down-regulated in the colonic mucosa, and the expression of the antimicrobial peptide Reg3g was also down-regulated, which implied the damaged colonic mucosal barrier with immunocompromised status. Finally, DCA-derived TDCA might promote the expression of Hnrnpa2b1 and enhance the stability and expression of TCF mRNA in the colonic mucosa, which subsequently strengthened the activity of the Wnt signaling pathway and promoted intestinal epithelial-mesenchymal transition (EMT). Therefore, DCA simulated bile reflux and induced CAG with the colonic mucosal lesion, which is accompanied by a damaged colonic mucosal barrier, mucosal immunocompromised status, and upregulating genes related to intestinal mucosal epithelial cell proliferation.

In summary, DCA simulated bile reflux to induce CAG and led to gut dysbacteriosis, mucosal barrier damage, and immune dysfunction. Firstly, excessive DCA promoted the abundance of gut *Enterococcus* and *Blautia*, which in turn involved the transformation of bile acids and exacerbated the cytotoxic DCA and LCA. Secondly, the cytotoxic DCA and LCA could damage the colonic mucosal barrier and lead to colonic mucosal lesions. Furthermore, abnormal metabolism could inhibit the antigen-presenting function and T-cell immune response. These comprehensive effects caused inflammatory colonic mucosal lesion and promoted the gene expression related to epithelial damage-repair (Fig. 7). The present data brought new insights into the biological mechanisms of CAG with colonic mucosal lesion, which provided potential intervention targets for the prevention and treatment of gastrointestinal comorbidities.

### Supplementary Information

Supplementary Information.

## Data Availability

The datasets presented in this study can be found in online repositories. The raw reads of fecal microbiota and intestinal mucosa transcriptome were deposited into the NCBI Sequence Read Archive (SRA) database, and the Accession Numbers are PRJNA1036012 and PRJNA1036011, respectively.
