# Supplementary material for: Deoxycholic acid inducing chronic atrophic gastritis with colonic mucosal lesion correlated to mucosal immune dysfunction in rats
Source: Sci Rep. 2024 Jul 9;14:15798. doi: 10.1038/s41598-024-66660-3 (PMC11233621; doi:10.1038/s41598-024-66660-3)
Supplement: Supplementary file 1 — Supplementary Information. [file 41598_2024_66660_MOESM1_ESM.docx]

**Supplementary figures and legends**


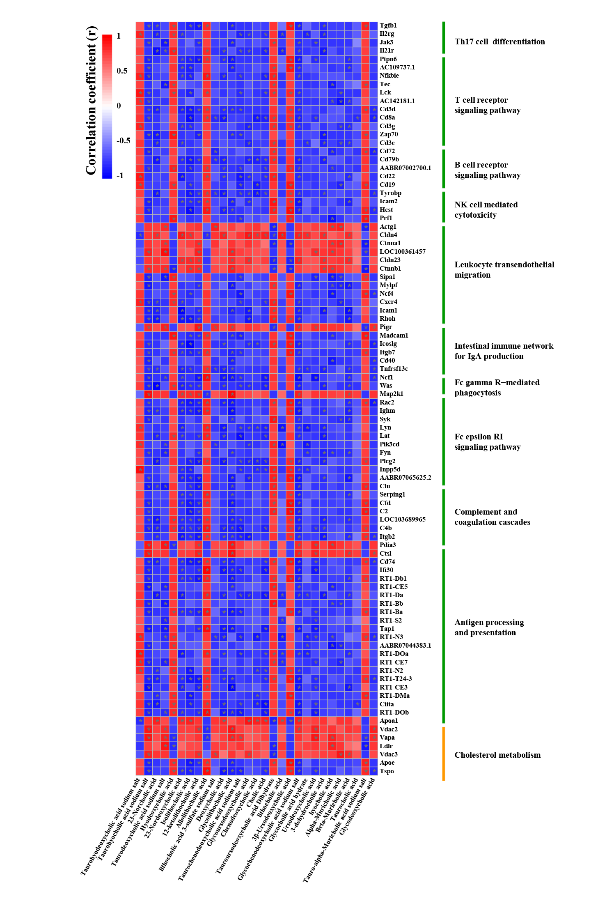


**Supplementary Figure 1.** Correlation analysis between DEGs and differential bacterial genera.

Spearman correlation analysis was used to construct a heatmap based on the colonic DEGs in 11 signaling pathways and differential bacterial genera. The red squares represent positive correlations, and the blue squares represent negative correlations. The asterisks mean significant correlations with absolute correlation coefficient (*r*) greater than 0.80 and *P* value less than 0.05.


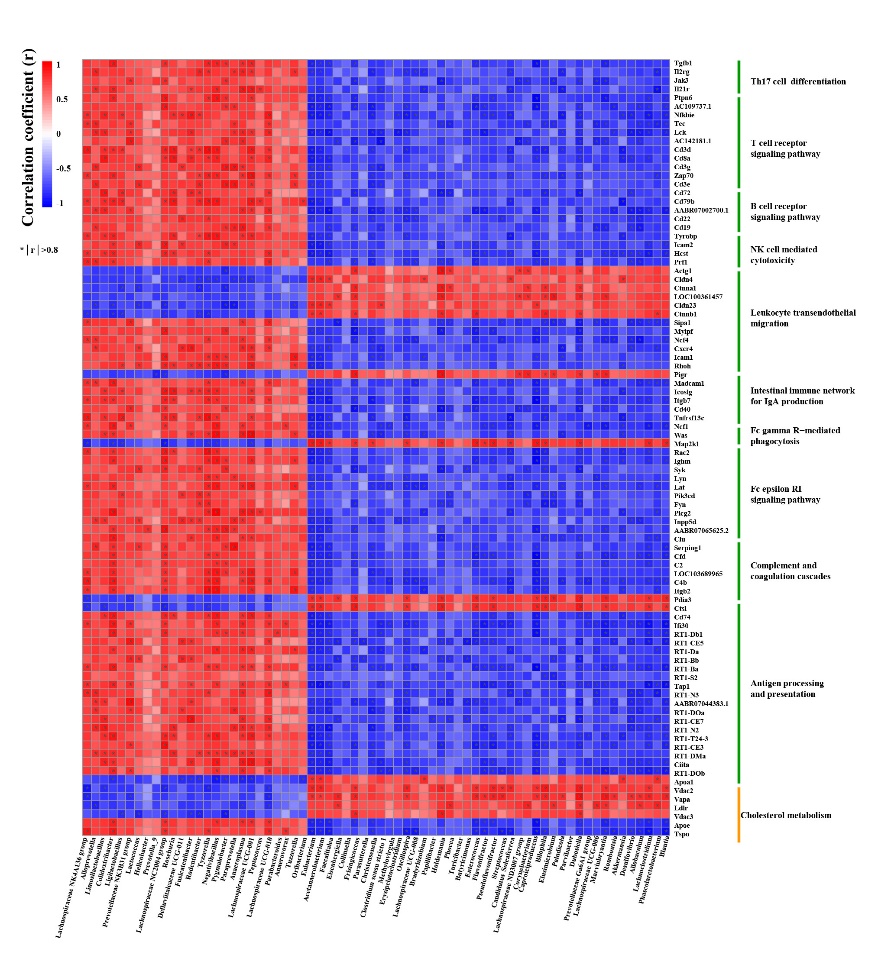


**Supplementary Figure 2.** Correlation analysis between DEGs and fecal BAs.

Spearman correlation analysis was used to conduct a heatmap based on the fecal BAs and the DEGs in 11 signaling pathways. The red squares represent positive correlations, and the blue squares represent negative correlations. The asterisks mean significant correlations with absolute correlation coefficient (*r*) greater than 0.80 and *P* value less than 0.05.

**Supplementary Table 1**. Sequence of primers used in RT-PCR

| Gene | Primers | Length |
| --- | --- | --- |
| Ciita | Forward: GACACTTCGGGCCGGGAGGTTGC  Reverse: AGCCGTGTGCCCAGGAAGGAGAGG | 123 |
| Cxcr4 | Forward: CCCGCCCTCCTCCTGACTAT  Reverse: GGCCTTGCGCTTCTGGTG | 225 |
| Hnrnpa2b1 | Forward: GCCGATATTGAGCTTCTTCCTAC  Reverse: TTTTTACAATCCTTCCTCCACAGT | 240 |
| Lrmp | Forward: CGATGCGGAGGAGAAGTGTGAC  Reverse: GAAGGACGCCCGCAGGTGAGTGA | 184 |
| Lck | Forward: GAAGGAGCGCCCAGAGGACAGG  Reverse: CCGCATGGATAGGGCACAAGAACT | 161 |
| Cd3d | Forward: CCCTGGCTGGTGTCATCATCA  Reverse: AAGACGGCTGTACTGGGCATCAT | 182 |
| β-actin | Forward: CTGGCTGGCCGGGACCTGACA  Reverse: ATTGCCGATAGTGATGACCTG | 219 |
